# Philanthropists among the London Poor

**Published:** 1895-11-23

**Authors:** 


### The Hospital

An Institutional Journal of

The Medical Sciences and Hospital Administration, with

AN EXTRA NURSING SUPPLEMENT.

Vol. XIX.—No. 478.] [Nov. 23, 1895.

### Philanthropists Among the London Poor.

Even in philanthropy natural selection is a prevailing law, and the survival of the fittest an inevitable result. The correspondence just published by the *Scientific Press* between Mr. Henry C. Burdett, Chairman of the Mansion House Committee of the Problem of the Poor, on the one hand, and Mr. C. S. Loch, Secretary of the Charity Organisation Society, on the other, shows how these natural forces work in things philanthropical, and gives the careful reader some indication of what the final issue is likely to be.

Mr. Loch is a keen critic, and an excellent expositor of his own views. Every friend of the Problem of the Poor scheme may feel happy when he reaches the close of the correspondence. He then knows one thing, if he knows nothing else, namely, that the most which can be said against the scheme of the Friendly Workers has been said, and said by a critic who knows exactly how to make every point tell; and yet when the worst has been said, in the most telling manner possible, it amounts to nothing at all. Mr. Loch says in effect that the Mansion House scheme is working largely on the lines of the Charity Organisation Society, and so far must be approved; but that in one or two comparatively unimportant details it is working on lines of its own, and so far must, by the Charity Organisation Society at any rate, be disapproved. More might have been feared from Mr. Loch—less was hardly to be expected. He will not bless, and he cannot ban.

Mr. George A. Spottiswoode, Chairman of the Committee of the House of Laymen, may not inappropriately be called in to act as discriminator and judge between the controversialists. Mr. Spottiswoode says that the Mansion House Committee "are on the right road towards solving the problem of the poor effectually." He states, as we think with peculiar wisdom, that "they have had the insight to perceive and the boldness to act upon the actual facts of the fabric of our English society; that they are endeavouring to make use of the forces actually at work instead of trying to dispense with them." The Committee of the House of Laymen, of which Mr. Spottiswoode is chairman, in a published report on the "Organisation of Philanthropic Efforts," declared that the Friendly Workers' movement, represented by the Mansion House Committee, is an instance where the co-operation of Christians and all religious denominations has been successfully secured for its work, so as to bring the whole power of religion to bear upon the social improvement of the people.

Here is the strength of the new movement. Mr. Benjamin Kidd, in his "Social Evolution," insists over and over again that the whole of Western civilisation, as it now exists, is based upon, springs from, and is permeated through and through by religious and ethical ideals. Western civilisation, he says, is not in any real and wide sense an intellectual, but a religious and an ethical development. If that be indeed so, the responsible heads of the Friendly Workers' scheme may have acted upon much more profound and far-reaching principles than they themselves were aware of when they founded this movement upon religion as a basis, and built it up by means of the representatives of every religious organisation in the metropolis.

The Friendly Workers' scheme, recognising as it does the forces of natural selection in every region of life, sets forth its own principles in logical form, tests them in definite areas in actual practice, and invites comparison with every other association which has for its object the solution of the problem of the poor. Those principles are now familiar to most of the philanthropic workers of London, and, notwithstanding Mr. Loch's claim on behalf of the Charity Organisation, they differ, in many respects fundamentally, from the principles upon which that organisation is based and worked. To begin with, the Friendly Workers' Association bases itself, for good or for ill, on religion. It has as a fundamental object the uniting of all religions in the common work of helping the helpless and the poor. In its very foundation and first principle, therefore, the Friendly Workers' Association differs from the Charity Organisation Society, which makes a boast of its undenominational character, that is, of its secular character. The Friendly Workers' Association, as its name implies, has the great object of enlisting all the altruistic feeling which is so widespread and so characteristic of modern times in the practical service of those who are but feebly equipped for the strenuous conflicts of modern life. In what sense does it here resemble the Charity Organisation Society? Work is the note of the Friendly Workers' method of help. Not work artificially created for the purpose, but work for which workers are being sought, and between which and the workless the association will act as the friendly medium of communication. Mr. Loch struck a very false note when he applied the word retrograde to the Labour Branch of the Mansion House Committee. If it be, then all work is retrograde, and all employers and employed are in combination to push civilisation down the steep hill of chaos and destruction. Knowledge, as against inquisition, is the Friendly Worker's guide in his activities. He takes his area of work, and makes it his business to know the business and history of every family in it, once and for all. That knowledge he keeps up by visitation. When, therefore, a crisis of want comes in any home in his area, he knows the home, the family, and the reason of the crisis, and can give the required help without painful inquisition or an hour's needless delay. How does this compare with the "inquisitions" and the "long delays" of the agents of the Charity Organisation Society?

In truth the correspondence between Mr. Loch and Mr. Burdett serves excellently well to bring out the deficiencies of the Charity Organisation Society, which are so fundamental, and which make it to day a bye-word in the mouths of the poor. On the other hand the more generous and brotherly aims of the Friendly Workers' Association are also brought into prominence, and its fitness for survival is rapidly being demonstrated in the areas of London in which for a longer or shorter period it has been so far tried. We are glad to note that the £1,500 which a generous donor placed at the disposal of the society the other day will be devoted to the establishment of a systematic training institution for Friendly Workers.

